# Maternal childbirth experience in induced and spontaneous labour measured in a visual analog scale and the factors influencing it; a two-year cohort study

**DOI:** 10.1186/s12884-020-03106-4

**Published:** 2020-07-21

**Authors:** Katti Adler, Leena Rahkonen, Heidi Kruit

**Affiliations:** Department of Obstetrics and Gynecology, University of Helsinki and Helsinki University Hospital, Haartmaninkatu 2, 00029 HUS Helsinki, Finland

**Keywords:** Induction of labor, Childbirth experience, Maternal satisfaction, Visual analogue scale, Operative delivery

## Abstract

**Background:**

Poor maternal childbirth experience plays a role in family planning and subsequent pregnancies. The aim of this study was to compare childbirth experiences in induced and spontaneous labor and to investigate the factors influencing the childbirth experience.

**Methods:**

This two-year cohort study included all women with term singleton pregnancies in cephalic presentation aiming for vaginal delivery at Helsinki University Hospital between January 2017 and December 2018. Maternal satisfaction in the childbirth experience was measured after delivery using a Visual Analog Scale (VAS) score. A low childbirth experience score was defined as VAS < 5. The characteristics and delivery outcomes of the study population were collected in the hospital database and analyzed by SPSS.

**Results:**

A total of 18,396 deliveries were included in the study, of which 28.9% (*n* = 5322) were induced and 71.1% (*n* = 13 074) were of spontaneous onset. The total caesarean delivery rate was 9.3% (*n* = 1727). Overall, 4.5% (*n* = 819) of the women had a low childbirth experience VAS score. The women who underwent labor induction were less satisfied with their birth experience compared to women with spontaneous onset of labor [7.5% (*n* = 399) vs. 3.2% (*n* = 420); *p* < 0.001]. Poor childbirth experience was associated with primiparity [OR 2.0 (95% CI 1.6–2.4)], labor induction [OR 1.6 (95% CI 1.4–1.9)], caesarean delivery [OR 4.5 (95% CI 3.7–5.5)], operative vaginal delivery [OR 3.3 (95% CI 2.7-4.0)], post-partum hemorrhage [OR 1.3 (95% CI 1.1–1.6)], and maternal infections [OR 1.7 (95% CI 1.3–2.4)].

**Conclusions:**

Poor childbirth experience was associated with labor induction, primiparity, operative delivery, and labor complications, such as post-partum hemorrhage and maternal infections. These results highlight the aspects of care for which patient experience may be improved by additional support and counselling.

## Background

The rates of induction of labor (IOL) are rising worldwide with currently almost every third labor being induced [1–3]. In Helsinki University Hospital, a tertiary care obstetric unit with 13,500 births annually, the rate of IOL is approximately 28%. The increasing rates in IOL may be explained by increasing maternal age, obesity, and medical conditions as well as by improved fetal monitoring and management practices. IOL is shown to improve perinatal outcomes without increasing the rate of caesarean deliveries [4].

An abundance of studies exist on the influence of pregnancy and labor on postnatal physical and mental health [5, 6]. Childbirth experience is influenced by a variety of health, social, and care factors [11]. Women undergoing IOL are less likely to be satisfied with their care and childbirth experience compared to women with spontaneous onset of labor [7, 8]. Women may be concerned about the impact of IOL on the fetus or themselves and more often express anxiety or report feelings of neglect, insufficient pain relief, plans not being followed, wasted effort, and disappointment if their labor induction is unsuccessful [9]. The progress of induced labor is one of the most important factors affecting overall maternal satisfaction [10]. Outpatient cervical ripening, sufficient patient information, and the active role of the woman improve the maternal experience [11, 12]. A poor childbirth experience plays a role in well-being after delivery, family planning, and subsequent pregnancies and deliveries. Thus, considering the increasing rates of IOL, optimizing the maternal childbirth experience in induced labor is important.

The aim of this study was to compare the maternal childbirth experience in induced and spontaneous labor and to investigate the factors influencing the childbirth experience.

## Methods

This retrospective cohort study included all women with live singleton pregnancies in cephalic presentation at or beyond 37 gestational weeks with the aim of vaginal delivery at Helsinki University Hospital between January 1st, 2017 and December 31st, 2018. The study protocol was approved by the institutional review board (IRB) of the hospital region (Helsinki and Uusimaa Hospital District Committee for Obstetrics and Gynecology) (nr. HUS/3172/2018 and HUS/54/2019). Due to the retrospective nature of the study, written informed consent was waived by the IRB according to national legislation (Medical Research Act 488/1999, Chap. 2 a [23.4.2004/295], Sect. 5 and 10a).

The primary outcome was the maternal childbirth experience, which was measured after delivery using the Visual Analog Scale (VAS) score [13]. The VAS is frequently used in pain measuring, but it has also been used for evaluating patient satisfaction, such as maternal satisfaction related to the birth experience [14–16]. The childbirth experience measurement was carried out according to the hospital policy by the treating midwife before discharging the patient from the post-partum ward after delivery. The patients were asked to rate their overall childbirth experiences on a scale from zero to 10, where zero stands for the most negative experience possible and 10 for the most positive experience possible. A VAS score of four or less was defined as a low score and a poor childbirth experience based on the local management recommendation to offer extra psychosocial support to women scoring a VAS of 0–4. The women who gave a low childbirth experience score were offered a consultation with a trained midwife or obstetrician according to the hospital management guideline.

The data on baseline characteristics and delivery outcomes of the study population were collected from the hospital database. The collected delivery parameters included the mode of delivery, post-partum hemorrhage (PPH) ≥ 1000 ml, grade III-IV perineal tear, placental retention, birth weight, Apgar score, umbilical artery blood gas values, and admission to the neonatal intensive unit (NICU). The correlation of maternal childbirth experience and the labor outcome parameters was included in the analyses.

Post-term pregnancy was defined as gestational age ≥ 41 weeks. Group B Streptococcus (GBS) was universally screened in all women by a rapid qualitative in vitro GBS test (Xpert® GBS, Cepheid, Sunnyvale, California, USA). Administration of prophylactic antibiotics was started for all GBS-positive women at the time of diagnosing ruptured membranes. Gestational diabetes was diagnosed by at least one pathological value with a two-hour oral glucose tolerance test. Failed induction was diagnosed after ruptured membranes and 12 hours of oxytocin administration without cervical change. Shoulder dystocia was defined as a delivery that required additional obstetric maneuvers to deliver the fetus after the head was delivered and gentle traction had failed.

The IOL was carried out through oral 50 mcg of misoprostol administered every four hours or a single 40–80 ml balloon catheter (Rüsch 2 way Foley Couvelaire tip catheter size 22 Ch, Teleflex Medical, Athlone, Ireland) retained for a maximum of 24 hours. When a Bishop score of ≥ 6 was reached, IOL was continued by amniotomy in case of intact amniotic membranes and oxytocin in the absence of spontaneous contractions. Oxytocin augmentation and continuous cardiotocography during labor were routinely used.

Statistical analyses were performed using the IBM SPSS Statistics for Windows, Version 26.0 (Armonk, NY, USA). Categorical variables were compared by the chi-square test and Fisher’s exact test when appropriate. Data with continuous variables were analyzed by a T-test when the data followed normal distribution and by a Mann-Whitney U test if the data did not follow normal distribution. Univariate and multivariate logistic regression analyses were performed to assess a relative risk for low VAS. Categorical variables were analyzed for odds ratios (OR) with a 95% confidence interval (CI). Adjusted odd ratios (OR) with 95% confidence intervals (CI) were calculated by modelling the data to control for possible confounding factors, as presented in Table 3. A *p*-value < 0.05 was considered statistically significant.

## Results

During the study period, 19,414 deliveries met the study criteria. The childbirth experience VAS score was missing in 5.2% (*n* = 1018) of the deliveries, leaving a total of 18,396 women included in the study. Of these, 28.9% (*n* = 5322) underwent IOL, and 71.1% (*n* = 13 074) were of spontaneous onset.

The mean maternal age was 31.8 (SD 5.0) years with the mean BMI 24.2 (SD 4.7) and the mean gestational age 40.2 (SD 1.2) weeks. The characteristics and labor outcomes of the study population are shown in Table 1. The women undergoing IOL were older, more obese, of more advanced gestational age, more often primiparous, or more often had IVF-pregnancy, gestational diabetes, and a history of previous CS (Table 1). The main indications for labor induction were post-term pregnancy (30.2%), prolonged pre-labor rupture of membranes (28.1%), gestational diabetes (10.6%), and hypertensive complications (7.2%). The overall CS rate was 9.3% (*n* = 1727). There were more caesarean deliveries (19.5% vs. 5.3%; *p* < 0.001) and labor complications, such as maternal infections, post-partum hemorrhage, and admission to neonatal care, among women whose labor was induced compared with those whose labor was of spontaneous onset (Table 1).

**Table 1:** The characteristics and delivery outcomes in women with induction of labor and spontaneous onset of labor (*n* = 18 396)

	Induced labor (*n* = 5322)		Spontaneous labor (*n* = 13074)		*p*-value
	**n**	**%**	**n**	**%**	
Age > 35 years	1613	30.3	3297	25.2	< 0.001
Height < 164 cm	1967	37.0	4651	35.6	0.01
BMI ≥ 30	1023	19.2	1165	8.9	< 0.001
IVF	313	5.9	525	4.0	< 0.001
Smoking	417	7.8	971	7.4	0.40
Primiparous	2799	52.6	5840	44.7	< 0.001
Post-term (≥ 41 weeks)	2259	42.4	3419	26.2	< 0.001
Gestational diabetes	1324	24.9	2505	19.2	< 0.001
Previous CS	462	8.7	834	6.4	< 0.001
Birth experience VAS-score < 5	399	7.5	420	3.2	< 0.001
Operative vaginal delivery	665	12.5	1515	11.6	0.08
Caesarean delivery	1040	19.5	687	5.3	< 0.001
Placental retention	140	2.6	190	1.5	< 0.001
Shoulder dystocia	22	0.4	40	0.3	0.25
Maternal infection	183	3.4	175	1.3	< 0.001
Sphincter injury	110	2.0	262	2.1	0.78
Maternal severe complication	7^a^	0.1	10^b^	0.1	0.27
Post-partum hemorrhage ≥ 1000 ml	775	14.6	1181	9.1	< 0.001
5-min Apgar score < 7	157	3.0	221	1.7	< 0.001
Umbilical artery pH < 7.05	74	1.5	147	1.3	0.58
NICU admission	345	6.5	661	5.1	< 0.001

*Abbreviations*: *BMI* body mass index, *IVF* in vitro fertilization

^a^ Relaparotomy *n* = 2, pulmonary embolism *n* = 1, sepsis *n* = 1, uterine rupture *n* = 3

^b^ Postpartum hysterectomy *n* = 1, uterine rupture *n* = 9

**Fig. 1:**
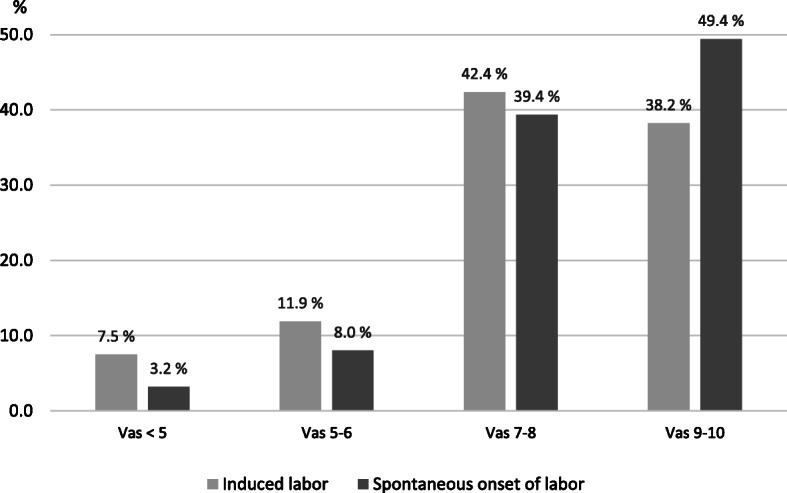
The distribution of birth experience VAS-score in induced and spontaneous onset of labor (*n*=18 396)

Figure 1 presents the distribution of the childbirth experience VAS score in the groups of induced and spontaneous labors. The mean (SD) VAS score was lower in women with induced labor compared to spontaneous onset of labor (7.7% vs 8.2%; *p* < 0.001). The VAS scores did not differ between the induction methods of a balloon catheter or misoprostol (VAS < 5 balloon catheter 7.5% vs. misoprostol 8.7%; *p* = 0.24). The proportion of a score of 9 − 10 was higher among women whose labor was of spontaneous onset (Fig. 1). Overall, 4.5% (*n* = 819) of the women gave a low childbirth experience VAS-score < 5. Primiparous women were more often less satisfied with their childbirth experiences compared with multiparous women [6.8% (*n* = 587) vs. 2.4% (*n* = 232); *p* < 0.001]. This was observed in the both groups of induced and spontaneous labors (Table 2). In both groups, women also more frequently gave a low childbirth experience VAS score following emergency CS or operative vaginal delivery compared to spontaneous vaginal delivery (Table 2).

**Table 2:** Low birth experience score (*n* = 819) presented as proportions of the parity and the mode of delivery groups

	Induced labor (*n* = 399)			Spontaneous labor (*n* = 420)		
	**n**	**%**	***p*-value**	**n**	**%**	***p*-value**
Parity			< 0.001			< 0.001
Nulliparous	285	10.2		302	5.2	
Multiparous	114	4.5		118	1.6	
Mode of delivery			< 0.001			< 0.001
Caesarean section	180	17.3		82	11.9	
Spontaneous vaginal	134	3.7		212	1.9	
Operative vaginal	85	12.8		126	8.3	

Women who had a low childbirth experience VAS score were more often obese, smokers, primiparous, post-term, more often had IVF-pregnancy, and underwent IOL (OR 3.0 95% CI 2.1–2.8) (Table 3). There were higher rates of CS (32.0% vs. 8.3%; *p* < 0.001, OR 5.2 [95% CI 4.4-6.0]) and operative vaginal delivery (25.8% vs. 11.2%; *p* < 0.001 OR 2.8 (95% CI 2.3–3.2)] among women who had a low VAS score compared to those with a VAS- score > 5 (Table 3). A low childbirth experience score was also associated with labor complications, such as placental retention, shoulder dystocia, maternal infection, sphincter injury, PPH, maternal severe complication, and NICU admission (Table 3). After the multivariable analysis was adjusted for factors presented in Table 3, only primiparity, labor induction, CS, operative vaginal delivery, maternal infection, and PPH remained significant (Table 3).

**Table 3:** Characteristics and delivery outcomes in women with low birth experience score compared to women with average or good birth experience (*n* = 18 396)

	VAS < 5 (*n* = 819)		Vas ≥ 5 (*n* = 17 577)		*p*-value	Unadjusted	Adjusted
	**n**	**(%)**	**n**	**(%)**		**OR (95% CI)**	**OR (95% CI)**
Age > 35 years	207	25.3	4703	26.8	0.35	0.9 (0.8–1.1)	0.9 (0.8–1.1)
Height < 164 cm	314	38.5	6304	36.1	0.161	1.1 (1.0–1.3)	0.9 (0.8–1.1)
BMI ≥ 30	133	16.2	2055	11.7	< 0.001	1.5 (1.2–1.8)	1.2 (1.0–1.5)
IVF	51	6.2	787	4.5	0.02	1.4 (1.1–1.9)	0.9 (0.7–1.3)
Smoking	79	9.8	1309	7.6	0.02	1.3 (1.0–1.7)	1.3 (1.0–1.6)
Primiparous	587	71.7	8092	45.8	< 0.001	3.0 (2.6–3.5)	2.0 (1.6–2.4)
Post-term (≥ 41 weeks)	338	41.3	5340	30.4	< 0.001	1.6 (1.4–1.9)	1.1 (1.0–1.3)
Induced labor	399	48.7	4923	28.0	< 0.001	2.4 (2.1–2.8)	1.6 (1.4–1.9)
Gestational diabetes	188	23.0	3641	20.7	0.12	1.1 (0.9–1.3)	1.0 (0.9–1.2)
Previous CS	59	7.2	1237	7.0	0.89	1.0 (0.8–1.3)	1.1 (0.8–1.5)
Caesarean delivery	262	32.0	1465	8.3	< 0.001	5.2 (4.4–6.0)	4.5 (3.7–5.5)
Operative vaginal delivery	211	25.8	1969	11.2	< 0.001	2.8 (2.3–3.2)	3.3 (2.7–4.0)
Placental retention	24	2.9	302	1.7	0.01	1.7 (1.1–2.6)	1.2 (0.7–1.9)
Shoulder dystocia	7	0.9	55	0.3	0.01	2.7 (1.2–6.0)	1.6 (0.7–3.9)
Sphincter injury	25	3.1	347	2.0	0.03	1.6 (1.3–2.4)	1.4 (0.9–1.2)
Maternal infection	64	7.8	294	1.7	< 0.001	5.0 (3.8–6.6)	1.7 (1.3–2.4)
PPH ≥ 1000 ml	174	21.5	1782	10.2	< 0.001	2.4 (2.0–2.9)	1.3 (1.1–1.6)
Maternal severe complication	5^a^	0.6	12^b^	0.1	< 0.001	9.0 (3.2–25.6)	3.0 (1.0–9.3)
NICU admission	80	9.8	926	5.3	< 0.001	2.0 (1.5–2.5)	0.9 (0.7–1.1)

^a^ Uterine rupture *n* = 2 (of which one underwent hysterectomy), relaparotomy *n* = 1, pulmonary embolism *n* = 1, sepsis = 1

^b^ Uterine rupture *n* = 10, hysterectomy *n* = 1, relaparotomy *n* = 1

## Discussion

In this two-year tertiary hospital cohort study of 18,396 women, a total of 4.5% of women had a low childbirth experience VAS score. Poor childbirth experience was associated with primiparity, labor induction, operative delivery, and maternal labor complications, such as PPH and infections. As the maternal childbirth experience may play an important role in family planning and subsequent pregnancies, assessing the factors influencing childbirth experiences is important. These results highlight the specific patient groups and aspects of care for which patient experiences could be improved by additional support and counselling.

In this study, primiparity was a significant risk factor for a low childbirth experience score. This was also observed in a previous survey study on more than 5,000 women in which multiparous women were more likely to have a positive experience than primiparous women [17].

Consistent with our results, IOL and labor interventions are associated with more negative childbirth experiences compared to spontaneous onset of labor [7, 8]. In a mixed-methods study on 5,333 women, delay in labor induction, delay in transfer to delivery ward, and delay in receiving pain relief were mentioned as the key themes for poor childbirth experiences [8, 18]. This may explain why some women with successful IOL and vaginal delivery in our study were not satisfied with their childbirth experiences. In addition, a lack of information and choice as well as feelings of disappointment, anxiety, and neglect have been discussed in previous studies [5]. These negative feelings are more likely to occur in cases of failed induction, prolonged labor, and other labor complications [15], which was also observed in our study in which labor complications, operative delivery, maternal infections, and PPH were associated with poor childbirth experiences. Furthermore, post-partum problems and length of post-partum hospital stay have been previously associated with negative childbirth experiences [11], which was also reflected in the current study.

In our study, women who had CS or operative vaginal delivery were less likely to have a positive childbirth experience compared to women with spontaneous vaginal delivery, which is in line with previous studies [8, 19]. Failed induction and prolonged labor have previously been reported as significant factors of a negative childbirth experience [9, 10]. This may explain some of our findings because failed induction and labor dystocia were common indications for CS in our study.

Neonatal care admission was not found to be a significant factor in childbirth experiences in some studies [10, 20, 21], while in others, it was found to be salient [10]. In our study, admission to NICU was not associated with a low childbirth experience score; however, maternal post-partum complications were associated with poor childbirth experiences in our study, which may partly be explained by maternal health issues, perhaps preventing an active role in the postnatal care of the infant and breastfeeding. Furthermore, the length of postnatal hospital stay and delay in recovery may have influenced interactions between the mother and the infant [11].

The major weaknesses of our study are the retrospective design and the childbirth experience VAS score being a subjective rating that may have been influenced by a variety of factors, such as individual variation of midwives discussing the birth and obstetric interventions and presenting the VAS scale. Furthermore, non-native speakers were involved in the study, so there may have been a lack of clarity involved in some cases. The VAS score is a narrow measurement for overall birth experience, and it may be influenced by several factors, such as delivery expectations, social status, support, labor analgesia, communication with medical staff, involvement in decision making, and the opportunity to discuss the labor after delivery. However, Larsson and colleagues have shown VAS to be suitable for evaluating the negative childbirth experience, and VAS has been validated for assessing birth experiences by comparing VAS with the Wijma Delivery Expectancy/Experience Questionnaire with a significant correlation between the two measurements [14, 16] According to the hospital protocol, the VAS assessment was performed 1–3 days after delivery, just before discharging the patient from the postpartum ward. The optimal time for evaluating a birth experience may be questioned, and the VAS score in our study may have been influenced by the short time interval from delivery and the initial positive feelings towards having a baby [22]. However, this is a large two-year cohort with more than nine of 10 women who delivered in a large tertiary care hospital having given the childbirth experience score, which adds to the value of the study. The authors acknowledge that investigating structured reasons and women’s perceptions of labor rather than relying on population characteristics and labor outcome data would improve these findings. We also regret not having the data on duration of labor. In addition, because the childbirth experience is influenced by a variety of health, social, and care factors, including data on long-term mental health, family planning, and social issues would be ideal.

## Conclusion

In conclusion, 4.5% of the women had a poor childbirth experience, which was associated with primiparity, labor induction, CS, and operative vaginal delivery as well as labor complications, particularly PPH and maternal infections. These results highlight the aspects of care in which patient experience could be improved by additional support and counselling. As pregnancy and childbirth may influence later health and family planning, structured studies on factors and interventions affecting the maternal childbirth experience are needed.

## Data Availability

The data that support the findings of this study are available from the Helsinki Uusimaa Hospital District but restrictions apply to the availability of these data, which were used under license for the current study, and so are not publicly available. Data are however available from the authors upon reasonable request and with permission of the Helsinki Uusimaa Hospital District.

## Abbreviations

IOL: Induction of labor
CS: Caesarean section
BC: Balloon catheter
RCT: Randomized controlled trial
IRB: Institutional review board
GBS: Group B Streptococcus
IVF: In-vitro fertilization
BMI: Body mass index

## References

[1] Vuori E, Gissler M. National Institute of Finland for Health and Welfare. Perinatal statistics: parturients, deliveries and newborns 2018.

[2] Zeitlin J, Mohangoo AD, Delnord M, Cuttini M (2013) The second European Perinatal Health Report: documenting changes over 6 years in the health of mothers and babies in Europe. J Epidemiol Community Health 67(12):983–5. 10.1136/jech-2013-203291 24052513

[3] Martin JA, Hamilton BE, Osterman MJK, Driscoll AK, Drake P (2018). Births: Final Data for 2017. Natl Vital Stat Rep.

[4] Mishanina E, Rogozinska E, Thatthi T, Uddin-Khan R, Khan KS, Meads C (2014). Use of labour induction and risk of cesarean delivery: a systematic review and meta-analysis. CMAJ.

[5] Nuutila M, Halmesmäki E, Hiilesmaa V, Ylikorkala O (1999) Women's anticipations of and experiences with induction of labor. Acta Obstet Gynecol Scand 78(8):704–9. 10468063

[6] Parratt J (2002) The impact of childbirth experiences on women's sense of self: a review of the literature. Aust J Midwifery 15(4):10–6. 10.1016/s1031-170x(02)80007-1 12593243

[7] Waldenstrom U, Hildingsson I, Rubertsson C, Radestad I (2004). A negative birth experience: prevalence and risk factors in a national sample. Birth.

[8] Henderson J, Redshaw M (2013a) Women's experience of induction of labor: a mixed methods study. Acta Obstet Gynecol Scand 92(10):1159–67. 10.1111/aogs.12211 23808325

[9] Gatward H, Simpson M, Woodhart L, Stainton MC (2010) Women's experiences of being induced for post-date pregnancy. Women Birth 23(1):3–9. 10.1016/j.wombi.2009.06.002 19647506

[10] Shetty A, Burt R, Rice P, Templeton A (2005) Women's perceptions, expectations and satisfaction with induced labour–a questionnaire-based study. Eur J Obstet Gynecol Reprod Biol 123(1):56–61. 10.1016/j.ejogrb.2005.03.004 15905017

[11] Henry A, Madan A, Reid R (2013). Outpatient Foley catheter versus inpatient prostaglandin E2 gel for induction of labour: a randomised trial. BMC Pregnancy Childbirth.

[12] Howard K, Gerard K, Adelson P, Bryce R, Wilkinson C, Turnbull D. Women's preferences for inpatient and outpatient priming for labour induction: a discrete choice experiment. BMC Health Serv Res. 2014; 14:330. Published 2014. doi:10.1186/1472-6963-14-330. 10.1186/1472-6963-14-330 PMC4128401 25073486

[13] Pham CT, Crowther CA (2003). Birth outcomes: utility values that postnatal women, midwives and medical staff express. BJOG.

[14] Larsson C, Saltvedt S, Edman G, Wiklund I, Andolf E (2011). Factors independently related to a negative birth experience in first-time mothers. Sex Reprod Healthc.

[15] Falk M, Nelson M, Blomberg M (2019). The impact of obstetric interventions and complications on women’s satisfaction with childbirth a population based cohort study including 16,000 women. BMC Pregnancy Childbirth.

[16] Wijma K, Wijma B, Zar M (1998). Psychometric aspects of the W-DEQ; a new questionnaire for the measurement of fear of childbirth. J Psychosom Obstet Gynaecol.

[17] Henderson J, Redshaw M (2013). Who is well after childbirth? Factors related to positive outcome. Birth.

[18] Leap N, Sandall J, Buckland S, Huber U (2010) Journey to confidence: women's experiences of pain in labour and relational continuity of care. J Midwifery Womens Health 55(3):234–42. 10.1016/j.jmwh.2010.02.001 20434083

[19] Rowlands IJ, Redshaw M (2012). Mode of birth and women’s psychological and physical wellbeing in the postnatal period. BMC Pregnancy Childbirth.

[20] Schytt E, Hildingsson I (2011). Physical and emotional self-rated health among Swedish women and men during pregnancy and the first year of parenthood. Sex Reprod Health.

[21] Heimstad R, Romundstad PR, Hyett J, Mattsson LA, Salvesen KA (2007) Women's experiences and attitudes towards expectant management and induction of labor for post-term pregnancy. Acta Obstet Gynecol Scand 86(8):950–6. 10.1080/00016340701416929 17653880

[22] Soet JE, Brack GA, DiIorio C (2003) Prevalence and predictors of women's experience of psychological trauma during childbirth. Birth 30(1):36–46. 10.1046/j.1523-536x.2003.00215.x 12581038

